# Pascal short-pulse plus subthreshold endpoint management laser therapy for diabetic macular edema: the “sandwich technique”

**DOI:** 10.1186/s40942-022-00381-5

**Published:** 2022-06-02

**Authors:** J. A. Cardillo, M. W. Rodrigues, R. C. Oliveira, A. M. V. Messias, R. Jorge

**Affiliations:** 1 Department of Ophthalmology, CRESEP- Eye Hospital public service, Araraquara, SP Brazil; 2 Department of Ophthalmology, Ribeirão Preto Medical School, University of São Paulo, 3900, Bandeirantes Avenue, Ribeirão Preto, SP 14049-900 Brazil

**Keywords:** Macular grid and focal laser, Short-pulse laser, Subthreshold laser, Diabetic macular edema

## Abstract

**Background:**

Diabetic macular edema (DME) is the main cause of visual loss in diabetic patients. Despite the use of anti-VEGF therapy as first-line treatment, there are many patients whose response to treatment is poor or transient at best. Sophisticated laser techniques have emerged aiming at low-intensity retinal damage, avoiding excessive heat that causes tissue necrosis and related collateral effects.

**Objective:**

To evaluate the effect of combined sublethal laser modalities from short-pulse duration (SPD) with endpoint management (EpM) subthreshold laser [named the “sandwich technique” (SWiT)] on central subfield thickness (CST) and best-corrected visual acuity (BCVA) in patients with DME.

**Material and methods:**

In this consecutive retrospective study, 37 patients (37 eyes) with center-involved (CI) DME were treated with SWiT laser therapy from April 2017 to June 2021. The technique consisted of a mean number of 200 (range number 50–400) SPD laser burns OCT-guided thickened area performed on the juxta- and perifoveal area 500 µm away from the foveal center, overlapping with a mean number of 1000 (range number 800–1200) EpM laser burns focused on 6 mm macular diameter area but saving 300 µm toward the foveal center. All patients underwent ophthalmological evaluations, including BCVA and CST measurement by spectral-domain optical coherence tomography (SD-OCT), before and after SWiT laser therapy. The mean follow-up time was 19.2 months (range 2–60 months).

**Results:**

Thirty-five out of 37 cases showed an improvement in CST and BCVA following treatment. At baseline, mean CST (µm) ± standard error (SE) and mean BCVA (logMAR) ± SE was 456.95 ± 37.00 and 0.71 ± 0.29, respectively. After a mean follow-up of 19.2 months, mean CST (µm) ± SE and BCVA (logMAR) ± SE were 272.09 ± 9.10 (p < 0.0001) and 0.54 ± 0.26 (p = 0.003), respectively. A statistically significant reduction in CST and improvement in BCVA was noted after laser therapy application. The anti-VEGF injection frequency was reduced during the mean 19.2 months of the study period.

**Conclusions:**

The novel “sandwich” laser therapy aid reduced CST and improved BCVA in this retrospective case series. Further prospective studies are warranted.

**Supplementary Information:**

The online version contains supplementary material available at 10.1186/s40942-022-00381-5.

## Introduction

In the next three decades, the prevalence of diabetes will more than triple universally, radically increasing the concern with this disease worldwide [1]. Diabetic macular edema (DME) is the leading cause of visual impairment in diabetic patients [2–4]. Formerly, in the 1980s, the Early Treatment Diabetic Retinopathy Study (ETDRS) revealed a substantial advantage of laser photocoagulation for the treatment of clinically significant DME, diminishing the incidence of moderate visual loss by approximately 50% after 3 years of follow-up [5].

The Diabetic Retinopathy Clinical Research network (DRCR.net) protocol V compared vision loss at 2 years among eyes with DME initially managed with aflibercept, laser photocoagulation (modified ETDRS), or observation. Eyes randomized to laser treatment were less likely to require aflibercept rescue therapy (p = 0.01) than eyes randomized to observation. Among randomized eyes with center-involved (CI) DME and good visual acuity, there was no significant difference in vision loss after 2 years [6].

The vascular endothelium growth factor (VEGF), originally deemed the vascular permeability factor, is a potent inducer of vessel permeability and macular edema [7]. Recent trials using anti-VEGF therapies for DME have shown significant results. Despite the use of anti-VEGF therapy as first-line treatment for center-involved DME, there are many patients whose response to therapy is poor or transient at best. In the RISE/RIDE trials, approximately 50% of patients failed to achieve a ≥ 15-letter gain in best-corrected visual acuity (BCVA), despite 2 years of monthly ranibizumab (0.5 mg) injections [8]. Additionally, some of these patients sustained persistent or worsening edema and/or vision loss despite treatment [9, 10].

The renowned DRCR.net study-protocol T demonstrated that aflibercept therapy led to superior outcomes in eyes with worse baseline acuity. However, laser rescue therapy was required for persistent DME defined based on protocol-specified criteria [11]. Over the course of 2 years, 41%, 64%, and 52% of the eyes treated with aflibercept, bevacizumab, or ranibizumab, respectively, received focal/grid laser therapy (p ≤ 0.01 for each pairwise comparison) [12]. Interestingly, this protocol revealed a high percentage of laser rescue therapy, pondering thoughts as a combined treatment study instead of single-drug regimens. Among two-thirds of the eligible Protocol T participants who completed a 5-year follow-up, mean VA improved from baseline to 5 years, without protocol-defined treatment after follow-up ended at 2 years [13].

**Fig. 1:**
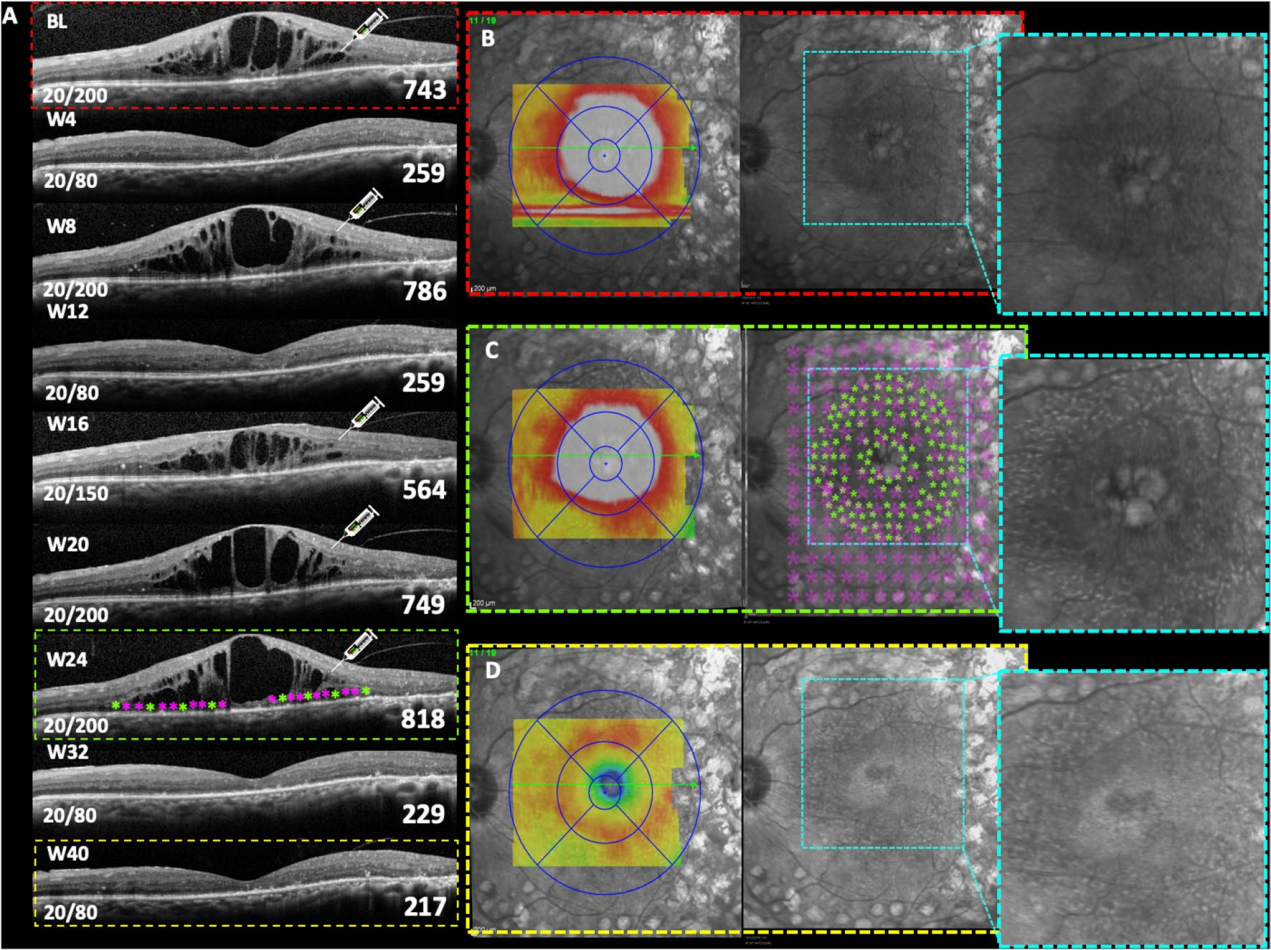
Patient with chronic DME and recently unsustained anti-VEGF response. **A** SD-OCT monthly follow-up of prn-IVA (*"pro re nata"* intravitreal aflibercept) therapy with associated SWiT laser therapy on week 24 after two consecutive unresponsive IVA injections. Anatomical SD-OCT image showing hyporreflective intraretinal cysts with mean CST (µm) and BCVA (Snellen) of approximately 743 and 20/200, respectively, at baseline. **B** Macular thickness map (dashed red rectangle) correlated with cystic hyper-reflectance edema on near-IR (dashed cyan square). **C** Twenty-fourth week of follow-up showing SD-OCT section [smaller dashed green rectangle with representative laser targets spots from green (SPD) and purple (EpM) asterisks] and the mean CST of 818 µm on the thickness map (bigger dashed green rectangle) and near-IR revealing hyper-reflectance dots (dashed cyan square) from recent SWiT laser therapy (SPD, green stars and EpM, light purple stars). **D** Forty-week follow-up revealing SD-OCT section (smaller dashed yellow rectangle) and the mean CST of 217 µm on the thickness map (bigger dashed yellow rectangle) after 8 weeks of combined IVA and SWiT laser therapy. Absent cystic hyper-reflectance edema showing ellipsoid band irregularities corroborating hyper-reflectance dots on near-IR (dashed cyan square). Visual acuity (BCVA of 20/80) stability since the 32nd week of follow-up

The Pascal (pattern scanning laser) device is a semiautomated laser that reduces procedural time by delivering multiple laser burns in a single application and allows for the controlled delivery of arrays with predetermined parameters. Muqit et al. [14] reported a significant retinal thickness reduction after 3 months using barely visible 10-ms Pascal laser burns. Also, the results from the study by Jain et al. [15] revealed a significant reduction in mean CST and improvements in mean BCVA scores after 4 months of short-pulse duration (SPD) Pascal laser usage. These results indicate a hypothetical biologically therapeutic effect or photorepair. Over time, lesions generated by the SPD laser tend to contract instead of expanding (Fig. 1D compared to Fig. 1C, dashed cyan square showing faded reflectance from near-infrared after 4 months of SPD laser therapy). Furthermore, the SPD laser procedure is safer since it uses, on average, 40% less energy per lesion compared to mETDRS treatment (3 vs. 5 mJ) [16].

**Fig. 2:**
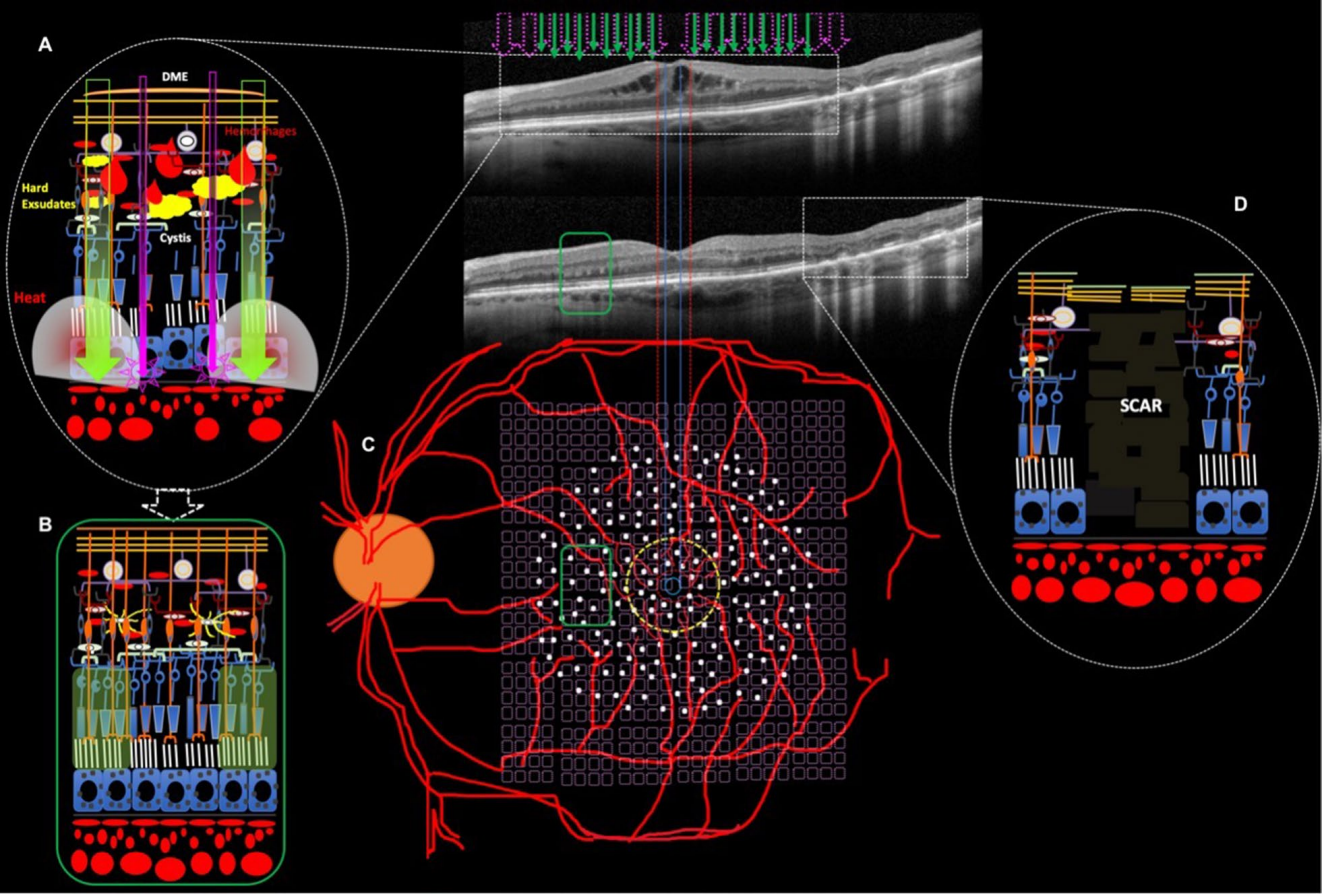
Schematic representation of laser display and anatomical responsiveness. **A** Representative retinal image section (dashed white oval) of photothermal effects producing retinal photorepair from SD-OCT image (dashed white rectangle) of retinal edema during SWiT laser therapy. **B** Representative retinal image section (green rectangle) from SD-OCT image of solved macular edema. **C** Representative vascular retina on posterior pole showing SWiT arrangement comprising barely visible marks spread 500 µm from the foveal center (SPD, white dots) and invisible marks (EpM, light purple dots) overlapping the SPD-treated area and reaching up to 300 µm from the foveal center. **D** Representative retinal image section (dashed white oval) of ETDRS laser scar from SD-OCT image (dashed white rectangle) performed in the past

**Fig. 3:**
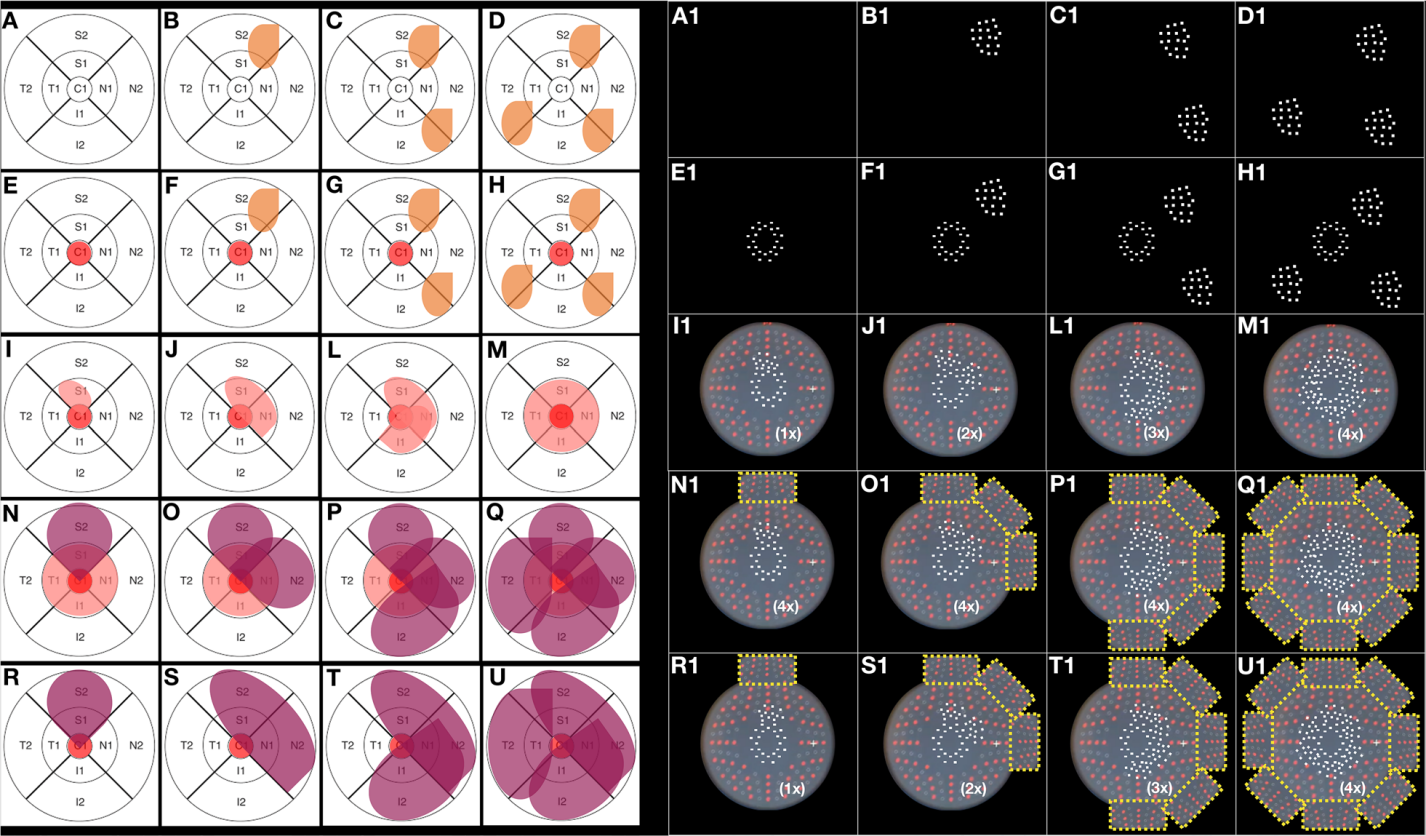
DME pattern and laser approach in OCT-guided technique. **A–A1** Regular CST ≤ 300 µm. **BB1–DD1** Extra-foveal DME. **E–E1** Center-involved (CI) DME. **FF1–HH1** Extrafoveal DME associated with CI-DME. **II1–MM1** Progressive inner subfield involvement associated with CI-DME. **NN1–QQ1** Progressive outer subfield involvement associated with the inner subfield and CI-DME. **RR1-UU1** Progressive inner-outer quadrant subfield involvement associated with CI-DME

Refined procedures have emerged aiming at a photorepairing effect using low-intensity power (with barely visible titration), short-time release, and high-density delivery onto the OCT-guided thickness area for the SPD laser modality (Fig. 3). The SPD and subthreshold laser modalities (e.g., micropulse diode laser or Pascal^®^-EpM) allow effective therapy with only sublethal thermal elevations, avoiding the excessive heat that causes white burns, tissue necrosis, and associated collateral effects (Fig. 2D) [17–19]. Our group proposed a combined Pascal laser modality: SPD *plus* EpM, as first described in the literature and denominated as the “Sandwich Technique” (SWiT), intending to add a flexible repair strategy to DME management (Fig. 2A–C).

## Methods

### Study design

The present study was conducted in compliance with the Declaration of Helsinki and was approved by the Research Ethics Committee of the Ribeirão Preto Medical School of the University of São Paulo (Protocol No.: 5.328.644). Data were collected in a retrospective fashion. The records of all patients attended at a public vitreoretinal subspecialty practice, who had undergone SWiT laser therapy for the primary diagnosis of DME, were reviewed, including 37 eyes from April 2017 to June 2021.

### Patient demographics

The following patient parameters were assessed: age, sex, hemoglobin A_1_c, hypertension (HTN) history, long-term diabetes history, previous pan-retinal photocoagulation (PRP), previous and current intraocular injection procedures, and diabetic retinopathy (DR) severity (Additional file 2: Table S1).

### Examination procedures

All patients underwent comprehensive ophthalmic examination comprising the BCVA test with an ETDRS vision chart, slit-lamp biomicroscopy, indirect ophthalmoscopy, near-infrared reflectance scanning laser ophthalmoscopy, fluorescein angiography, and spectral-domain optical coherence tomography (SD-OCT) (Spectralis; Heidelberg Engineering, Germany).

### Patient eligibility and exclusion criteria

The inclusion criteria were as follows: (1) 18 years old or above with diabetes mellitus (type 1 or 2); (2) BCVA between 0.3 logMAR (Snellen equivalent: 20/32) and 1.3 logMAR (Snellen equivalent: 20/400), and (3) center-involved DME with CST > 300 µm by SD-OCT, despite cataract surgery performed at least 4 months prior. As for the exclusion criteria, the following were considered: any evidence of vitreomacular traction by SD-OCT; no history of vitreoretinal surgery; no severe capillary nonperfusion on fluorescein angiography; no other ophthalmic disease other than cataracts.

### Treatment regimen

All treatments were provided using a Pascal^®^ photocoagulation unit, which uses a 532-nm frequency-doubled solid-state green laser source. An expert physician performed the laser therapy on all patients. The SWiT laser therapy involved two photothermal stimulation methods: (1) sublethal SPD and (2) subthreshold EpM, which were applied in all cases. Briefly, in this novel technique, the titration for the SPD laser method (10 ms; 100-µm spot size) was performed in approximately 1–3 single shots with adjusted power to cause a light gray barely visible burn near the emerging optic disc vessels outward regarding the superior or inferior nasal vascular arcades (Additional file 3: Table S2). After titration, the single focal SPDs were manually selected on a touchscreen display with 100% of titrated power, and initial individual shots were precisely placed 500 µm from the foveal center, 360º around the CST area (CST > 300 µm, Fig. 3E–E1). Eventually, the extra-foveal thickened area may require an associated segmental grid SPD guided by the OCT thickness map (Fig. 3F–H and F1–H1). Similarly, the non-center-involved DME may regard segmental grid SPD shots guided by the OCT thickness map based on physician judgment (Fig. 3B–D and B1–D1). The full-grid SPD pattern (1. 100 radius of up to 6 mm diameter), which includes 56 spots simultaneously triggered on mode A (red dots on Pascal grid pattern standby, Fig. 3I1–U1) or 56 spots simultaneously triggered on mode B (gray dots on Pascal grid pattern standby, Fig. 3I1–U1), was used depending on inner subfield OCT thickness map involvement. Up to four full-grid SPD multiple shot triggers were applied proportionally to up to four compromised inner subfield regions, respectively (Fig. 3I–M and I1–M1). In addition, focal and segmental grid SPDs were still required to access strictly juxtafoveal and parafoveal areas (Fig. 3I1–U1, white dots), respectively (because of a lack of spots inside the inner 1. 100 radius Pascal pattern ring). In the case of extensive DME in outer subfield areas, the segmental grid SPDs were available to cover extra location (Fig. 3 N–U and N1–U1, dashed yellow square). After SPD therapy, the titration for the subthreshold EpM laser (15 ms; 200-µm spot size) technique was performed with approximately 1–3 single shots with power adjusted to cause a light gray barely visible burn, which was located near emerging optic disc vessels outward regarding the superior or inferior nasal vascular arcades. After the titration step, the EpM energy setting was lowered to 30% and applied using a 16-shot square grid pattern with 0.25-mm spacing diameter apart, sweeping (one or two times) the posterior pole at a 3000-µm radial extension from the foveal center until up to 300 µm and overlapping the SPD laser-treated areas (Fig. 2C, dashed light purple square grid and Additional file 3: STable 2) [20, 21]. Retinal microaneurysms were not treated focally.

### Statistical analysis

Data were collected in a retrospective manner based on medical chart review. Statistical analyses were conducted using the single-arm univariate Student T-test, with statistical significance corresponding to a p-value of 0.05, using the JMP 10.0.0 software (2010; SAS Institute Inc, Cary, North Carolina, USA). The mean BCVA and CST measured after SWiT laser therapy were compared to the mean baseline values of the two parameters using multiple analysis of variance (MANOVA) for repeated measures.

## Results

Between April 2017 and June 2021, 37 subjects (mean age of 65.8 ± 8.2 years; 50% women) were enrolled in this study. Ninety percent of the patients (33/37) had undergone at least 5 months of follow-up after SWiT laser therapy. The mean follow-up period was 19.2 months. Two new cases (5.4%) had recently been diagnosed with DME (< 6 months) and SWiT laser therapy was applied combined with anti-VEGF therapy as initial treatment. The majority of patients (35/37, 94.6%) had chronic DME (> 6 months of duration). Most of them had already received different DME therapies in the past (anti-VEGF and/or TAAC and/or ETDRS focal/grid laser). The mean number of anti-VEGF injections required was approximately 2 (± 1) throughout all the follow-up visits after SWiT laser therapy.

### Effect of treatment on retinal thickening [Additional file 4: Graph S1B]

The mean CST (µm) ± SE was 456.95 ± 37.00 at baseline and decreased significantly to 272.09 ± 9.10 after associated SWiT laser therapy (mean follow-up of 19.2 months; p < 0.0001). Seventy-five percent of the eyes (28/37) underwent a decrease in CST of 100 µm or higher.

### Effect of treatment on visual acuity [Additional file 4: Graph S1A]

The mean baseline BCVA (log-MAR) ± SE was 0.71 ± 0.29 (Snellen equivalent: 20/100) and improved significantly to 0.54 ± 0.26 (Snellen equivalent: 20/70) after associated SWiT laser therapy (mean follow-up of 19.2 months; p = 0.003). Approximately one-third (12/37, 30%) of the subjects showed no improvement in BCVA compared to baseline values. In contrast, (25/37, 70%) had a BCVA improvement of 5 letters or more.

### The T-test and single variable correlations between BCVA and CST at baseline and after SWiT laser therapy

A significant correlation (*r* = 0.64) was found between baseline BCVA and BCVA after SWiT laser therapy. There was also a significant correlation (*r* = 0.52) between CST and BCVA after related SWiT laser therapy. Multivariable analysis demonstrated that only 2 variables (CST after and BCVA prior to SWiT laser therapy) influenced BCVA after SWiT laser treatment. Therefore, CST before SWiT laser therapy was not significantly correlated to BCVA after SWiT laser therapy.

## Discussion

The “sandwich technique” received its name because of joined laser modalities that resulted from an overlap of sublethal SPD with subthreshold EpM laser therapy (Fig. 2A–C). We decided to apply denser laser shots (SPD) based on the OCT thickness map, according to the elaborated OCT-guided strategy shown in Fig. 3. Meanwhile, the subthreshold laser (EpM) was applied diffusely over the posterior pole (Fig. 2C).

In fact, with sublethal low-intensity threshold techniques (i.e., SPD) that produce only low and confined thermal elevations (Fig. 2B, green rectangular box, sort of “chickenpox” or “woodpecker” style), there is very little lateral spread of heat (Fig. 2A) from the retinal pigment epithelium (RPE) spots directly targeted by the laser. In theory, the clinical response could be related to the laser-induced activation of cytokine expression and upregulation of matrix metalloproteinase (MMP)-3 in the retina, promoting subsequent cellular alterations that correct the pathological imbalance and stop the spread and migration of the RPE cells at the edges of the lasered site [22–25].

As previously determined, the enhanced expression of the heat shock protein (HSP-70) in the retina begins at a laser power of approximately half of the RPE damage threshold. Once laser-retinal interactions trigger HSP activation, an initiation step in a cascade of reparative phenomena improves RPE function, retinal autoregulation, acute reparative inflammation, reduced markers of chronic inflammation, and immunomodulation (Additional file 1: Fig. S1) [26].

Ideally, subthreshold photostimulation (i.e., EpM) results in laser spots that are not visible under any light microscope [27]. It has been suggested that subthreshold photocoagulation helps increase the permeability of the RPE at early stages and promotes the discharge of effusion in areas of edema. In later stages, RPE repair promotes metabolism and water discharge from the retina to the choroid [28]. Moreover, a lower risk level of damage to the retinal nerve fiber layer (RNFL) and reduced inflammatory reactions with visual function preservation are guaranteed with this technique [29]. Some clinical studies have concluded that subthreshold micropulse diode laser photocoagulation could improve visual acuity and alleviate DME [30].

Despite the lack of studies comparing subthreshold low-energy micropulse exposures (exhaustively studied since the 1990s) [31] and the modern subthreshold Pascal-EpM technique, it can be hypothesized, theoretically, that both nondestructive regimens may have analogous efficiency as nondamaging photorepairing laser modalities. It is relevant to clarify that in SPD (10-ms duration) at 100% energy levels (barely visible threshold), the photoreceptors and RPE are selectively targeted, leaving the inner retina intact. These lesions heal over 2 months, reestablishing the photoreceptor layer and local synapses between migrated photoreceptors and preserved bipolar cells [32]. Momentary disruption of the ellipsoid band suggests that each hyperreflective vertical mark may consist of coagulated photoreceptor elements and Müller cells within the OPL (Fig. 2B, green rectangle). Localized proliferation within the apical RPE with no morphological alterations within the inner retina correlates with reported histopathological work [33]. Over time, lesions created by shorter-duration lasers tend to contract instead of expanding [34]. Evidence of a therapeutic effect of short-term duration laser therapy for DME treatment has been published [14, 15]. Also, with the advent of the Pascal laser, multiple barely visible laser lesions can be applied safely and with a decreased risk of retreatment of a particular area [35, 36].

In this real-life study, a statistically significant reduction in CST (p < 0.0001) and a significant improvement in BCVA (p = 0.003) were observed after SWiT laser treatment for DME. The results obtained herein highlight the importance of sublethal laser modalities in managing DME. Pei-Pei et al. [37] showed results from a prospective randomized study comparing two Pascal laser modalities: subthreshold (EpM) versus threshold (SPD) laser therapy. There were no significant differences between groups regarding BCVA (p = 0.428) and CST (p = 0.399) outcomes at 6 months of follow-up. Both groups showed significant differences in mean CST at 6 months of follow-up compared to baseline (p < 0.05). Meanwhile, at 6 months, the threshold group did not present significant differences in mean BCVA compared to baseline (p = 0.065). However, the subthreshold group did (p = 0.035).

The absence and slight presence of thermal spread from subthreshold EpM and barely visible threshold SPD, respectively, should be compensated for with a higher density of applications to effectively treat thickened OCT-guided areas. The density optimization strategy plays a key role as a flexible repair plan for the treatment of DME under subvisible laser protocols [38]. Laser spot distribution (SPD modality) is planned based on CST maps according to thickened-retina distribution so that the diverse anatomical arrangement of the edema is treatable with individual density allocation (Fig. 3) and complementary EpM subthreshold laser are optimized according the DME anatomical characteristics and visual function. Thinner recent DMEs (< 400 µm) with low thickened-retina extension (central subfield and up to two OCT-map inner subfields) and less disorganized retinal layers with better BCVA (> 20/50 Snellen) suggest a favorable response to lower (SPD) and moderate (subthreshold EpM) shot densities. In contrast, thicker longstanding DMEs (≥ 400 µm) with high thickened-retina extension (central subfield and more than two OCT-map inner subfields) and haphazard full retinal layers with worse BCVA (< 20/50 Snellen) may indicate a better response to high densities of combined modalities: SPD and EpM. We believe that higher rates of threshold SPD in unhealthy cells potentially force lasting incompetent RPE/Glial cells to survive (“defibrillation effect”) or stimulate surrounding boundary viable cells for photoreceptor rearrangement and/or migration. Meanwhile, less functionally and structurally impaired retinal cells or even normal adjacent RPE cells may be more suitable for high proportions of subthreshold EpM for effectively alleviating DME.

A recent review published clinically relevant findings after 2 years of DRCR.net Protocol T use, in which potential future directions for persistent DME were addressed [39]. Refining the role of focal/grid laser treatment as a supplement to anti-VEGF for DME management was one of the discussed issues. As we know, the (m)ETDRS approach is still currently suggested in most well-known DME trials; however, it is not encouraged by laser experts presently [20, 21, 25, 36–38].

**Fig. 4:**
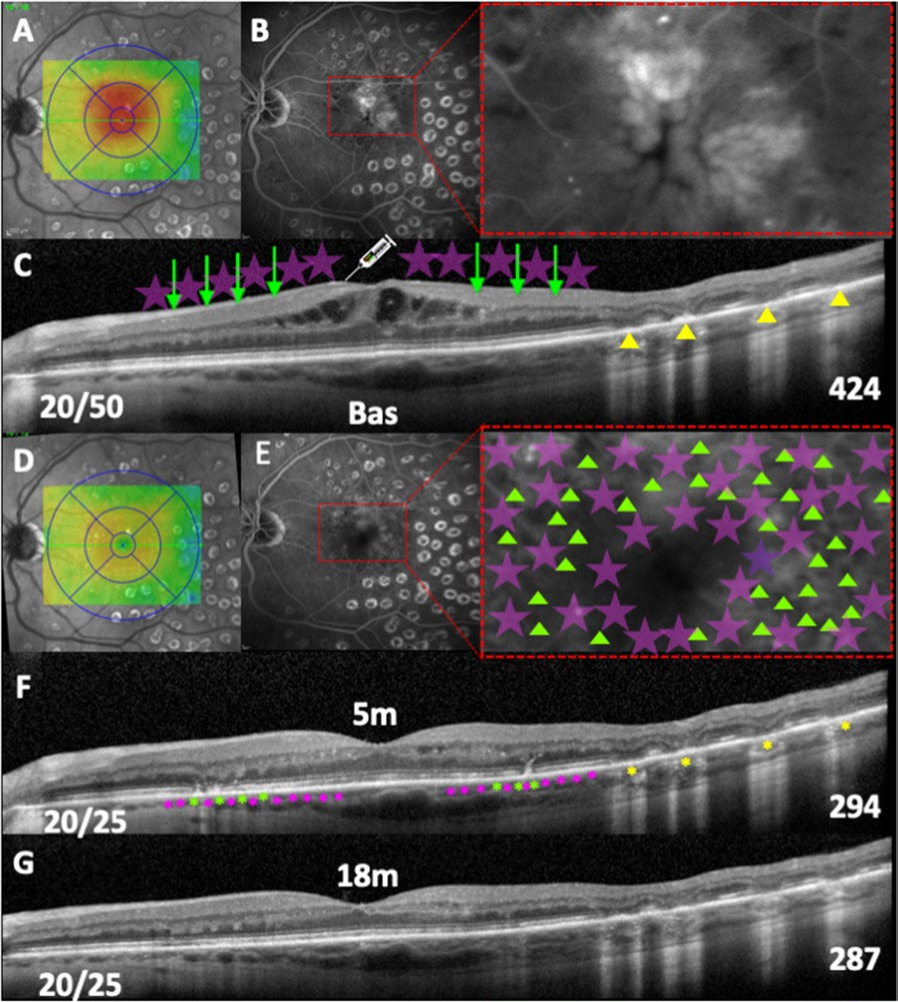
Patient with chronic and recurrent DME that required anti-VEGF therapy. **A** CST map overlying near-IR image revealing diffuse CI-DME. **B** Late phase of fluorescein angiography showing cystoid hyperfluorescent leaking (dashed red rectangle). **C** Baseline SD-OCT showing hyporreflective inner retinal cysts and atrophic outer retinal lesions located temporally to the fovea from previous ETDRS laser therapy (yellow arrowheads). The CST measured 424 µm. Illustrative IVR (intravitreal ranibizumab) injection combined with SWiT laser therapy (SPD, green arrows and EpM, light purple stars). Baseline BCVA (Snellen) was 20/50. **D** Five months after SWiT laser therapy with CST map showing an absence of DME. **E** Late phase of fluorescein angiography without leaking (dashed red rectangle) and presence of tiny hypofluorescent dots spread widely on the macular area, as a result of the SPD laser array (green arrowheads). Also, representative invisible EpM laser targets (light purple stars). **F** SD-OCT image of a five-month resolved macular edema showing hyperreflective dots in the outer retina due to photothermal cell activation from the SPD laser pattern (green asterisks) and subthreshold anatomical representative EpM targets (pink asterisks). The atrophic outer retinal lesions located temporally to the fovea from previous ETDRS laser therapy were maintained (yellow asterisks). Interestingly, the CST decreased to 294 µm. **G** Eighteen months later, the SD-OCT still showed a stable CST of 287 µm, and correlated visual acuity improvement (Snellen BCVA of 20/25) was obtained since the 5-month follow-up visit

In real-life conditions, most DME patients undergo chronic treatment and sustain consecutive follow-up visits. One study reported that approximately one in four patients with non-proliferative diabetic retinopathy, who had DME, did not return for follow-up for at least 1 year after anti-VEGF injection [40, 41]. Agarwal et al. [42] identified the following aspects as risk factors for noncompliance: non-white individuals, lack of bilateral treatment, and poorer glycemic control. Our study showed an extended-duration effect of combined laser therapy in the current DME patients, as shown, for example, in Fig. 4, in which an 18-month-old dried edema can be observed.

**Fig. 5:**
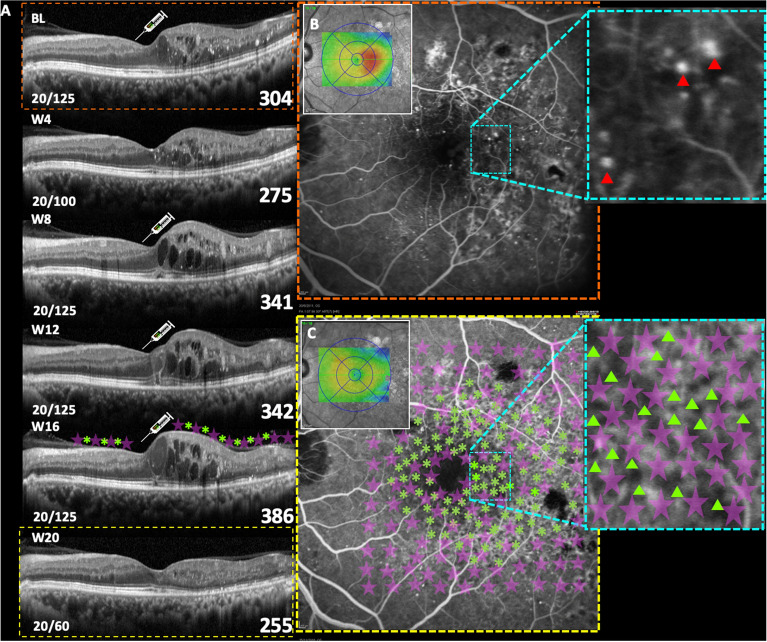
Patient with chronic DME and recently unsustained anti-VEGF response. **A** SD-OCT 20-week monthly follow-up of prn-IVA therapy and associated SWiT laser therapy on week 16 after two consecutive unresponsive IVA injections. Anatomical SD-OCT image with mean CST (µm) of 304 and visual BCVA (Snellen) of approximately 20/125 at baseline. **B** Baseline intermediate phase of fluorescein angiography (dashed orange square) revealing hyperfluorescent microaneurysms (red arrowheads) correlated with thickened edema (dashed cyan square) on the thickness-map (white-lined square). **C** Intermediate phase of fluorescein angiography (dashed yellow square) after a 20-week follow-up showing hypofluorescent density spots (green arrowheads) related to SPD laser shots and subthreshold anatomical representative EpM targets (light purple stars). Absence of edema (dashed cyan square) on the corresponding thickness map area (white-lined square) and undetectable fluorescein angiography of inactive microaneurysms. After the 20-week follow-up, visual improvement (Snellen BCVA of 20/60) correlated with the anatomical CST decrease (255 µm) on the SD-OCT image after 4 weeks of SWiT therapy

Associated SWiT therapy (Fig. 5) leads to fewer follow-up visits and decreases the burden costs for long-term treatment, as revealed in a 24-week Protocol T post hoc analysis, where   it was shown that extending the anti-VEGF loading dose from 3 to 6 injections requires investing €5882.77 (8 injections), €10,091.03 (14 injections), and €10,198.59 (30 injections) per additional responder patient (3-month nonresponders and 6-month responders) to aflibercept, ranibizumab, and bevacizumab, respectively [43].

The present study had some design limitations as a retrospective study, including a relatively small sample size and a lack of macular visual function examinations, such as contrast sensitivity and microperimetry. In addition, a more extensive prospective and randomized study protocol would provide further valuable insight into the effectiveness and reliability of this new strategy explored by SWiT laser therapy. The lack of trials using sophisticated techniques is still scant in the researcher community. Difficulties including high-investment devices, extensive physician training, and time-consuming procedures are considerably discouraging.

In good hands, inherent advantages from appropriate laser techniques can prevent the excessive number of intravitreal injections, particularly regarding patient convenience and treatment cost-effectiveness. The proposed SWiT laser treatment for retinal anatomical and visual photorepair may offer a promising alternative for enhanced beneficial effectiveness, raising doubt regarding the ancient mETDRS photocoagulation protocol. Avoiding archaic techniques that include thermal retinal injury photocoagulation, specialists have published advanced practices aimed at maximizing clinical effectiveness and enabling a finer control of the photothermal effects induced at the RPE level [44].

This retrospective study took into account various real-life cases. The idea of combining refined sublethal techniques, such as the novel SWiT laser method by using an OCT-guided model, revealed promising results in several types of DME worthwhile for future prospective studies.

## Supplementary Information

**Additional file 1: Figure S1.** **A** Representative image of DME. **B** Highly magnified RPE cell activated through SWiT laser photostimulation and intranuclear heat shock factor (HSF-1) stimulation to generate HSPs (heat shock proteins), with subsequent anti-apoptotic factor (Bcl2) formation and inflammation factor (TNF-alpha) depletion.

**Additional file 2: Table S1.** Baseline characteristics.

**Additional file 3: Table S2.** Titration and SWiT laser therapy configuration.

**Additional file 4: Graph S1.** **A** Mean BCVA (logMAR) at baseline (A1) and after SWiT laser therapy (A2); **B** Mean CST (µm) at baseline (B1) and after SWiT laser therapy (B2).

## Data Availability

The authors had full access to all the data in the study and take responsibility for the integrity of the data and the accuracy of the data analysis.

## Abbreviations

DR: Diabetic retinopathy
DME: Diabetic macular edema
VEGF: Vascular endothelium growth factor
SPD: Short-pulse duration
EpM: Endpoint management
SWiT: Sandwich technique
CST: Central subfield thickness
BCVA: Best-corrected visual acuity
CI: Center-involved
SD-OCT: Spectral-domain optical coherence tomography
SE: Standard error
ETDRS: Early treatment diabetic retinopathy study
DRCR.net: Diabetic retinopathy clinical research network
HTN: Hypertension
PRP: Pan-retinal photocoagulation
RPE: Retinal pigment epithelium
MMP: Matrix metalloproteinase
RNFL: Retinal nerve fiber layer
OPL: Outer plexiform layer
