# Supplementary figures and images for: Pascal short-pulse plus subthreshold endpoint management laser therapy for diabetic macular edema: the “sandwich technique”
Source: Int J Retina Vitreous. 2022 Jun 2;8:32. doi: 10.1186/s40942-022-00381-5 (PMC9161489; doi:10.1186/s40942-022-00381-5)

A

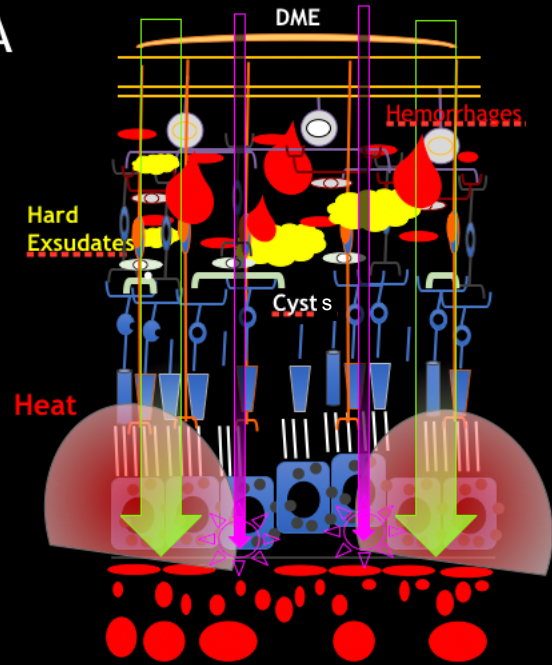

B

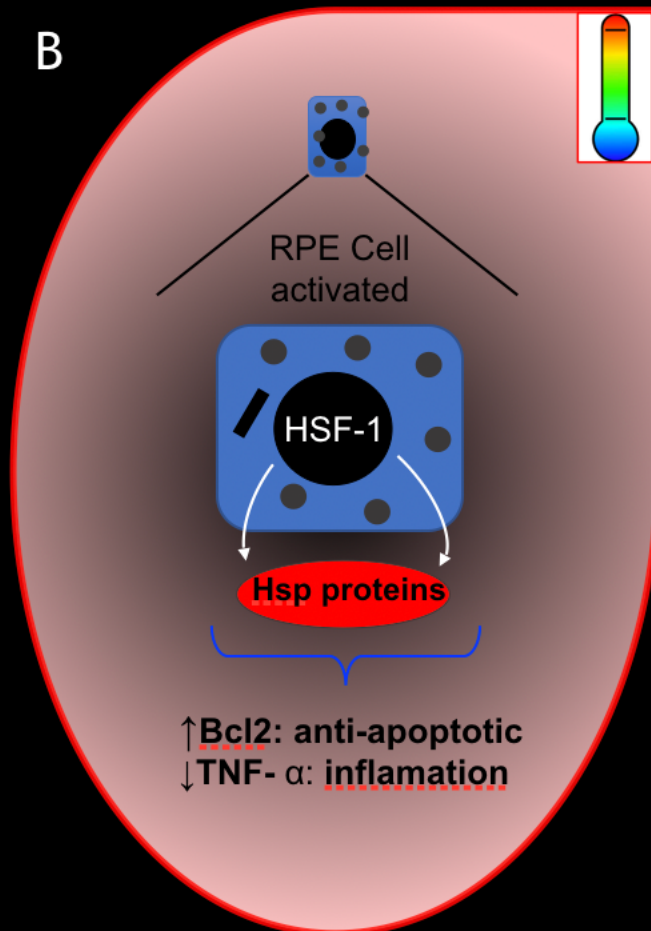

Supplement: Supplementary file 1 — Additional file 1: Figure S1. A Representative image of DME. B Highly magnified RPE cell activated through SWiT laser photostimulation and intranuclear heat shock factor (HSF-1) stimulation to generate HSPs (heat shock proteins), with subsequent anti-apoptotic factor (Bcl2) formation and inflammation factor (TNF-alpha) depletion. [file 40942_2022_381_MOESM1_ESM.pdf]

**A**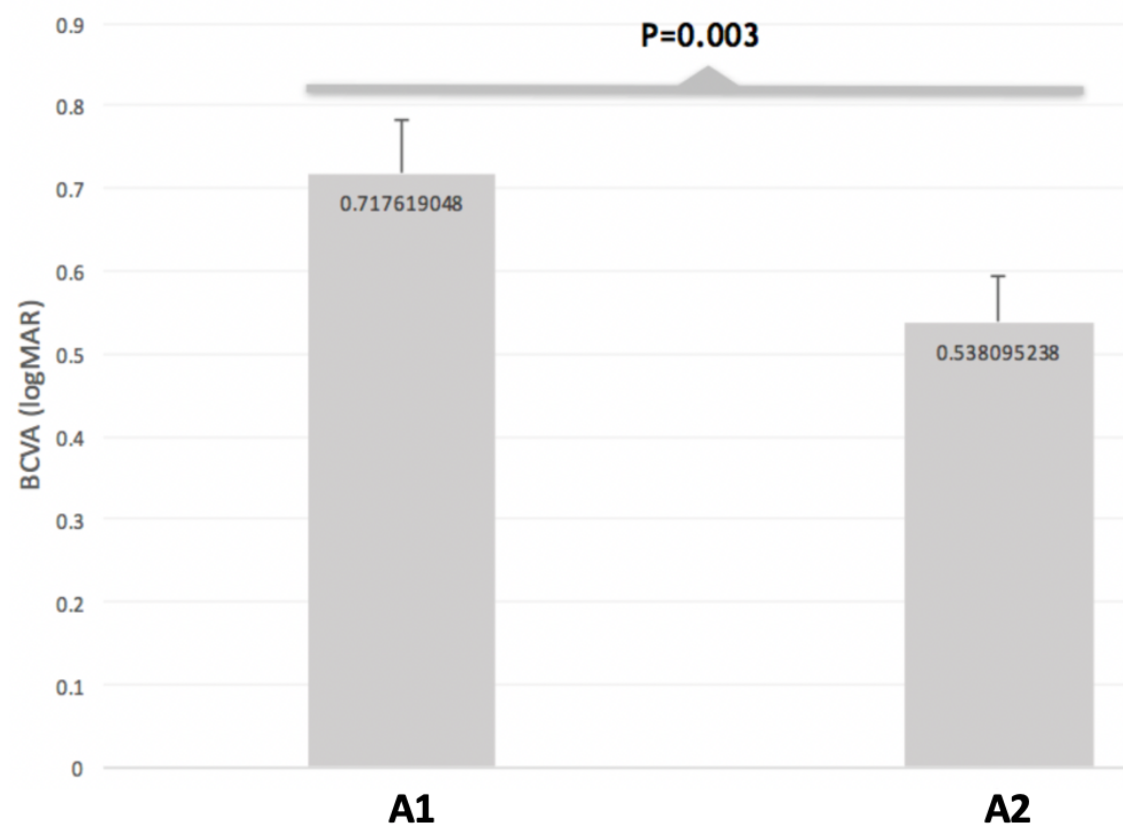**B**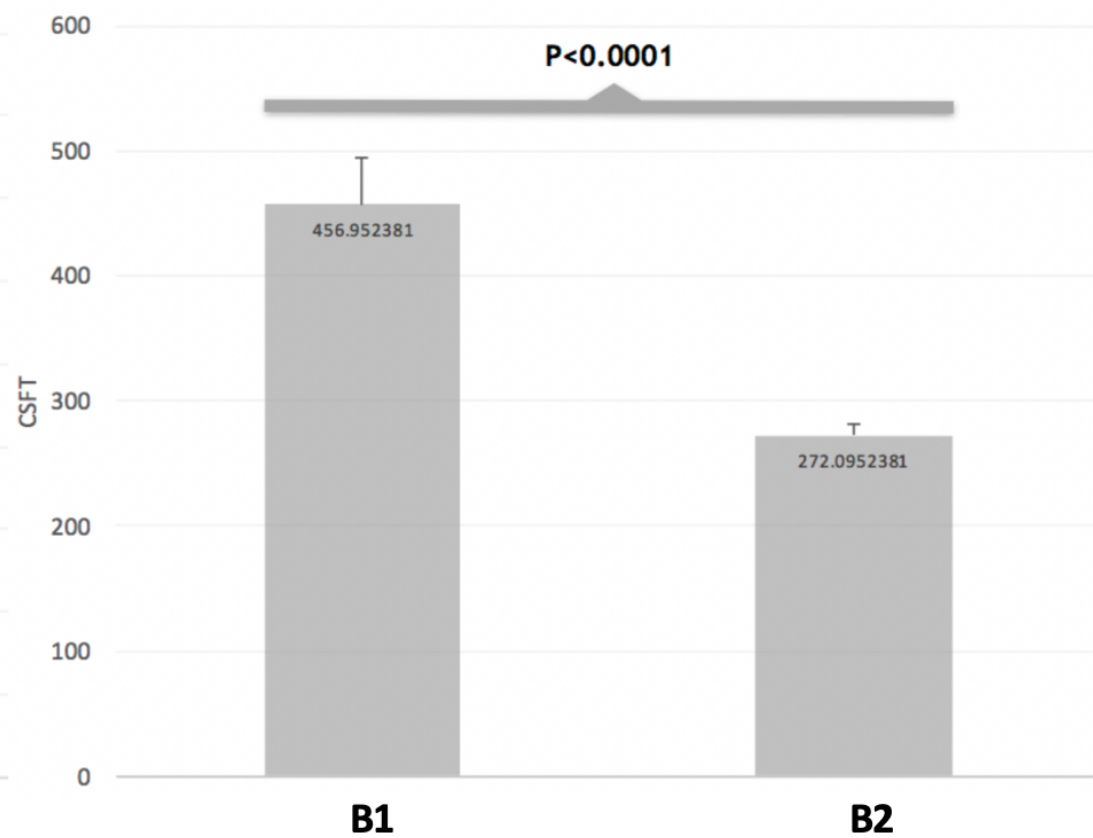

Supplement: Supplementary file 2 — Additional file 2: Table S1. Baseline characteristics. [file 40942_2022_381_MOESM2_ESM.pdf]
