# Supplementary material for: Pascal short-pulse plus subthreshold endpoint management laser therapy for diabetic macular edema: the “sandwich technique”
Source: Int J Retina Vitreous. 2022 Jun 2;8:32. doi: 10.1186/s40942-022-00381-5 (PMC9161489; doi:10.1186/s40942-022-00381-5)
Supplement: Supplementary file 3 — Additional file 3: Table S2. Titration and SWiT laser therapy configuration. [file 40942_2022_381_MOESM3_ESM.pdf]

Table 1

| Baseline Characteristics | |
|---|---|
| Age (years)(mean ± SD) | 65.8 ± 8.2 |
| Sex (female)(%) | 50 |
| Duration of Diabetes (years) | 17.3 ± 5.5 |
| Pseudophakic (%) | 50 |
| Treatment regimen: insulin (%) | 60 |
| HbA1c (mean ± SD) | 10.4 ± 1.1 |
| Proliferative Diabetic Retinopathy (%) | 52 |
| Chronic macular edema (> 6 months) (%) | 94.6 |
| Mean follow-up (months) | 19.2 |
