# Supplementary material for: Pascal short-pulse plus subthreshold endpoint management laser therapy for diabetic macular edema: the “sandwich technique”
Source: Int J Retina Vitreous. 2022 Jun 2;8:32. doi: 10.1186/s40942-022-00381-5 (PMC9161489; doi:10.1186/s40942-022-00381-5)
Supplement: Supplementary file 4 — Additional file 4: Graph S1. A Mean BCVA (logMAR) at baseline (A1) and after SWiT laser therapy (A2); B Mean CST (µm) at baseline (B1) and after SWiT laser therapy (B2). [file 40942_2022_381_MOESM4_ESM.pdf]

Laser titration				Laser treatment			
Mark		Duration (ms)	Spot (µm)	Power (mW)		Location	Number
*SPD	(Barely visible)	10	100	100% from titration (mW)	(Barely visible)	OCT-guided thickened area till 500µm from the foveal center	~50-400
**EPM	(light gray-yellowish)	15	200	30% from titration (mW)	(invisible)	6mm macular diameter area spread but saving 300µm toward the foveal center	~800-1200
Placement				Pascal shot modalities (manually selected on screen-monitor) and combined strategies			
near the main large optic disc vessels inward regarding the superior and inferior nasal vascular arcades;				**Focal** SPD: individual shots initially performed 360° around and 500µm from the foveal center on CST*** map<br>**Segmental grid** SPD: selected multiple spots in extrafoveal areas targeted in the OCT-guided thickness area<br>**Full grid** SPD pattern: selected multiple spots disposed 360° around the foveal center, which simultaneously received high-density shots<br>**Squared grid EPM:** 4x4 square grid placed on the extensive macular area overlapping the SP-therapy site			

* Pascal short pulse duration

**Pascal endpoint management

***Center subfield thickness
